# Lipid raft involvement in signal transduction in cancer cell survival, cell death and metastasis

**DOI:** 10.1111/cpr.13167

**Published:** 2021-12-22

**Authors:** Borui Li, Yi Qin, Xianjun Yu, Xiaowu Xu, Wenyan Yu

**Affiliations:** ^1^ Department of Pancreatic Surgery Fudan University Shanghai Cancer Center Shanghai China; ^2^ Department of Oncology Shanghai Medical College Fudan University Shanghai China; ^3^ Shanghai Pancreatic Cancer Institute Shanghai China; ^4^ Pancreatic Cancer Institute Fudan University Shanghai China

**Keywords:** CD44, Fas/CD95, IGF‐I/PI3K/Akt signalling, lipid rafts, VEGF/VEGFR2

## Abstract

Lipid rafts are cholesterol‐ and sphingolipid‐enriched specialized membrane domains within the plasma membrane. Lipid rafts regulate the density and activity of signal receptors by compartmentalizing them, promoting signalling cascades that play important roles in the survival, death and metastasis of cancer cells. In this review, we emphasize the current concept initially postulated by F. Mollinedo and C. Gajate on the importance of lipid rafts in cancer survival, death and metastasis by describing representative signalling pathways, including the IGF system and the PI3K/AKT, Fas/CD95, VEGF/VEGFR2 and CD44 signalling pathways, and we also discuss the concept of CASMER (cluster of apoptotic signalling molecule‐enriched rafts), coined, originally introduced and further advanced by F. Mollinedo and C. Gajate in the period 2005–2010. Then, we summarize relevant research progress and suggest that lipid rafts play important roles in the survival, death and metastasis of cancer cells, making them promising targets for cancer therapy.

## Graphical abstract

In this review, we focus on the importance of lipid rafts in cancer survival, death and metastasis by describing representative signalling pathways, including the IGF system and the PI3K/AKT, Fas/CD95, VEGF/VEGFR2 and CD44 signalling pathways, and we also discuss the concept of CASMER on the basis of predecessors study. Then, we summarize relevant research progress and suggest that lipid rafts play important roles in the survival, death and metastasis of cancer cells, making them promising targets for cancer therapy.

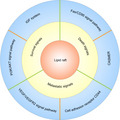

## 1 INTRODUCTION

The cell membrane consists of a lipid bilayer interspersed with different kinds of proteins. The heterogeneous distribution of certain types of lipids leads to the formation of different compartmentalization structures in which different types of proteins are selectively enriched or excluded. These dense and stable membrane domains are called lipid rafts, whose ordered compartmentalization structures are contiguous with the surrounding disordered lipid layers, reflecting the heterogeneity of cell membrane structures. In 1997, Simon and Ikonen proposed the lipid raft hypothesis, which extended the fluid mosaic model of the cell membrane proposed by Singer and Nicolson.^1^ According to the definition presented by the Keystone Symposium of Lipid Rafts and Cell functions in 2006, lipid rafts are small, heterogeneous and highly dynamic lipid domains in the cell membrane that are rich in sphingolipids and cholesterol.^2^ In the early 2000s, Mollinedo and Gajate found that lipid rafts were critical for the regulation of cell death mediated by death receptors,^3^, ^4^, ^5^ and proposed lipid rafts as hubs for the modulation of cell life and death, and for processes critical for cancer development and treatment,^3^, ^4^, ^5^ thus advancing lipid rafts as a target for cancer therapy.^6^, ^7^, ^8^, ^9^, ^10^

According to their different molecular hydrophilicities, sphingolipids are classified into sphingomyelin, cerebroside and ganglioside categories.^11^ Among these types, sphingomyelin and gangliosides are the main sphingolipids in lipid rafts.^12^ Fatty acid chains in the rafts tend to be densely packed, forming ordered lipid platforms that float in the phospholipid bilayer.^13^ In the plasma membrane, cholesterol increases the order of other lipids in the membrane, enhancing membrane fluidity and the lipid diffusion rate, regulating membrane permeability, ensuring mechanical coherence and preventing membrane leakage.^14^ Therefore, cholesterol is essential in the formation of lipid rafts. In addition, cholesterol is thought to serve as insulation between the carbon‐hydrogen bonds of sphingolipids and dynamic glue that keeps the components of the lipid raft together.^15^ The close interaction between sterols and sphingolipids in lipid rafts makes them insoluble in cold non‐ionic detergents, which are used to separate lipid rafts via density gradient centrifugation.^16^, ^17^, ^18^

Altering cholesterol levels or administrating agents that degrade sphingolipids in the cell membrane is a common method of disrupting lipid rafts and studying their function; the common reagents for these experiments are statins, which are used as treatments for hypercholesterolaemia.^19^ In some clinical studies, simvastatin alone or in combination with other chemotherapeutic agents has been shown to significantly improve treatment outcomes and reduce mortality in patients with certain types of cancer.^20^, ^21^

Lipid rafts can be classified into the flat type and invaginated type. Invaginated lipid rafts have a concave configuration, and their key component is caveolin. Three isoforms constitute the caveolin family: caveolin‐1, caveolin‐2 and caveolin‐3. Caveolin‐1 and caveolin‐2 are widely expressed in epithelial cells, and caveolin‐3 is highly expressed in striated and smooth muscle cells.^22^, ^23^ Compared with the invaginated type, flat lipid rafts have smaller volume and maintain a typical flat and orderly structure, and the flotillin protein is an indispensable component for its structure and functions.^24^

In general, lipid rafts are transient and heterogeneous in nature, serving as a framework for receptors and associated signalling molecules.^25^

**FIGURE 1:**
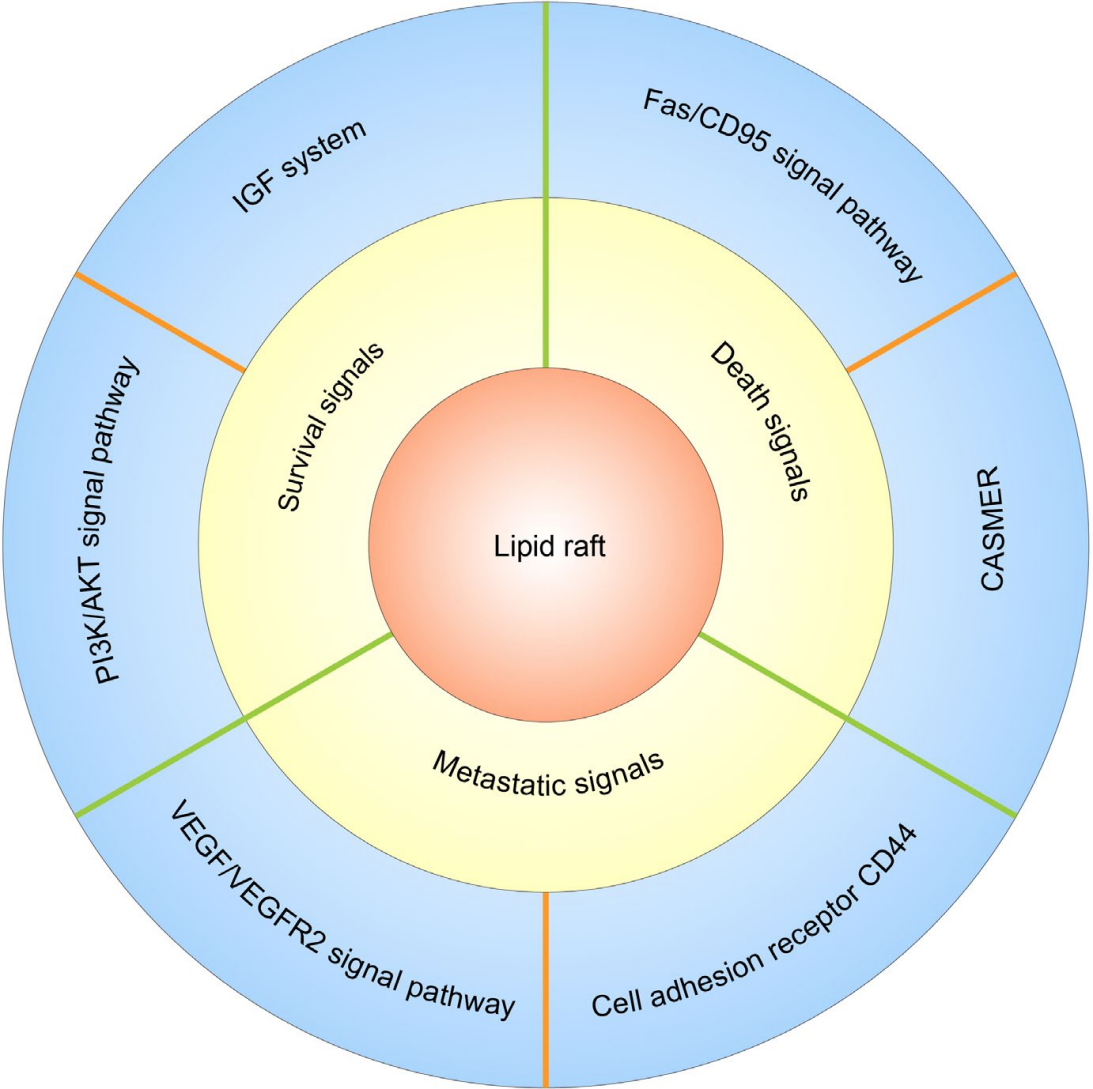
Graphical abstract(Figure inspired by Mollinedo and Gajate^26^)

The cell membrane plays an important role in cellular function regulation, particularly signal transduction. As a recruitment platform for signalling proteins, lipid rafts can selectively and dynamically recruit or exclude certain signalling proteins, kinases and phosphatases in response to stimuli inside and outside the cell by changing their size and composition and protecting related proteins from degradation, thus effectively promoting the interaction between proteins and cell signal transduction.^19^, ^26^, ^27^ Specifically, proteins undergo oligomerization and supramolecular aggregation in lipid rafts,^10^, ^26^, ^28^ and their physical distribution regulates their accessibility to effector molecules; in this way, the lipid raft is a centralized platform for specific receptors that are activated by ligand binding. In addition, lipid rafts can protect signalling complexes from the effect of non‐raft inhibitory proteins. This function of lipid rafts allows the isolation of proteins that can activate or inactivate certain pathways, facilitating or inhibiting downstream signal transduction.^10^, ^26^, ^28^ In the cancer field, many signalling pathways related to survival and proliferation have been shown to be connected with lipid rafts, such as the IGF system and PI3K/Akt pathway.^26^, ^29^ In addition, it has also been shown that some death receptors critical to the apoptosis signalling pathway need to be transported to and/or recruited by lipid rafts, among which CD95 is the most representative.^4^, ^5^, ^6^, ^7^, ^26^, ^30^, ^31^ Some studies have shown that lipid rafts may also be related to tumour drug resistance. For example, radiation can enhance the integrity of lipid rafts in lung cancer cells, which may be benefit for tumour cells that acquire radiation resistance.^32^ In addition, many reports have shown that lipid rafts can play an important role in cancer metastasis by regulating a series of signalling pathways related to cell adhesion and migration, such as the CD44 signalling pathway.^32^ This review summarizes the important role of lipid rafts in the transduction of survival signals, death signals and metastatic signals in cancer (Figure 1).

## 2 LIPID RAFTS AND SURVIVAL SIGNALS IN CANCER CELLS

Lipid rafts are widely present in the plasma membrane of eukaryotic cells and play important roles in cell survival. It has been reported that a large number of signal transduction processes occur in lipid rafts. Specifically, lipid rafts are closely related to carcinogenesis and cancer cell survival. According to Simons and Toomre,^19^ lipid rafts are closely related to cell survival signals transmitted through EGFR, the IGF system, neurotrophic factors, H‐ras, Akt, etc. Long before the concept of lipid rafts was proposed, cholesterol, one of its main components, was found to accumulate in malignant lesions.^26^, ^33^ Follow‐up studies showed that cholesterol levels in tumour cells are higher than those in normal cells.^34^ Further research showed that lipid rafts in cancer cells had higher cholesterol levels than those in normal cells.^35^ All these studies indicate the important role of lipid rafts, particularly the level of cholesterol enrichment in lipid rafts, in the development of cancer cells. Many growth factor systems and signalling pathways related to lipid rafts play important roles in tumour cell survival, among which IGF has been widely studied, and it has been proven that its overexpression and overactivation are crucial to tumour development.^26^, ^29^ The interactions between the IGF system or the downstream PI3K/Akt pathway and lipid rafts in cancer cell survival are described in the following sections.

## 3 LIPID RAFTS IN THE IGF SYSTEM

Insulin‐like growth factors (IGFs) are mitotic factors that have been shown to play key roles in regulating the proliferation, differentiation and apoptosis of various tumour cells, as well as in tumour angiogenesis.^36^, ^37^ IGFs include insulin and its structurally similar insulin‐like growth factor‐1 (IGF‐1) and IGF‐2. These growth factors bind to a pair of homologous tyrosine kinase receptors, namely, insulin receptor (IR) and insulin‐like growth factor‐1 receptor (IGF‐1R); these 2 receptors share 60% homology, and both contain 2 transmembrane and intracellular domains with tyrosine kinase activity (β‐subunit, 95 kDa) and two extracellular ligand domains (α‐subunit, 135 kDa). In addition, 6‐phosphate mannose/IGF‐2R (M6P/IGF‐2R) has been shown to be an IGF receptor unrelated to IGF/IR and IGF/IGF‐1R. The main IGF form is a complex consisting of IGFs and their carrier proteins, namely, IGF‐binding proteins (IGFBPs). Six kinds of IGFBPs have different affinities for IGF‐1R,^38^, ^39^ and they regulate IGFs transport, extracellular matrix deposition, receptor binding and degradation, which regulates receptor bioavailability.^40^

**FIGURE 2:**
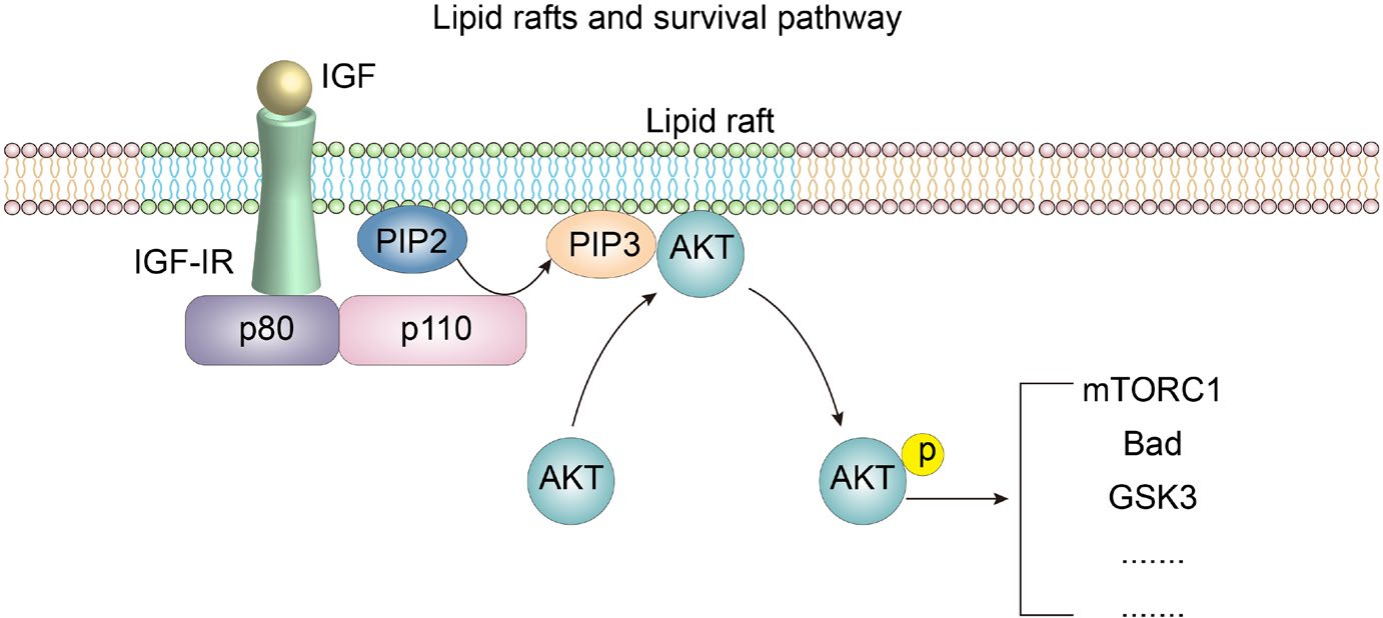
Lipid rafts and survival pathways(Modified from formers’ studies^26^, ^29^)

After ligand binding, IR and IGF‐1R are phosphorylated on tyrosine residues coupled with related pathways, including the PI3K/Akt pathway and MAPK pathway, thereby promoting cell survival and proliferation.^41^ In addition, IR and IGF‐1R can bind to their corresponding ligands, and cross‐binding between them has been identified, but the affinity of the crosslinked ligands and receptors is much weaker than the affinity between receptors and their specific ligands.^41^ After ligand binding, IGF‐1R activates intracellular tyrosine kinases and recruits and phosphorylated intracellular insulin receptor substrate SRC homology collagen (Shc) and other adaptor proteins; these substrate proteins bind to sh2 to stimulate various intracellular signalling cascades, one of which is a key pathway, the class I PI3K/Akt pathway. Activation of this pathway triggers many key functions of the IGF system, such as promoting cell proliferation and division (Figure 2) and inhibiting proapoptotic stimulation.^42^, ^43^ However, it has been reported that IGF‐1 conferred a protective effect on colorectal cancer cells against TNF‐α‐induced apoptosis and that this protective effect was gradually lost upon cholesterol depletion and restored with exogenous cholesterol supplementation.^44^ This result was most likely achieved through the function of lipid rafts.

With the deepening of research on the IGF signalling system and the function of lipid rafts in signal transduction, the relationship between the IGF signalling system and lipid rafts in the development of tumours has received increasing attention. A study by Huo et al.^45^ showed that IGF‐1R in 3T3‐L1 preadipocytes was localized in lipid rafts. IGF‐1R‐mediated signalling in 3T3‐L1 preadipocytes can be blocked by destroying lipid rafts through the consumption of cellular cholesterol with cholesterol‐binding reagents β‐methyl cyclodextrin or filipin. In their study, inhibition of caveolin expression by RNAi did not affect the number of lipid rafts, IGF‐1R localization in lipid rafts or signal transduction. Cholesterol‐binding agents blocked the signal transduction effects, suggesting that the lipid raft itself, not the recruitment of signalling molecules by caveolin, mediates IGF‐1R signalling.^45^, ^46^ In some tumours, IGF‐1R is redistributed in lipid rafts, thus activating downstream pathways and enhancing an antiapoptotic effect on tumour cells. For example, in MGC803 and BGC823 human gastric cancer cells, TRAIL death receptors not only induce apoptosis but also promote IGF‐1R migration to lipid rafts, thus activating survival signalling pathways to antagonize TRAIL‐induced apoptosis.^47^, ^48^ Furthermore, the distribution of IGF‐1R inside and outside lipid rafts is closely related to the survival and apoptosis of tumour cells. Remacle‐Bonnet et al.^44^ showed that IGF‐1R expression in colorectal cancer cells was significantly higher in non‐lipid raft domains on the plasma membrane during cholesterol depletion, and its apoptosis‐promoting effect was inhibited. In MCF‐7 human adenocarcinoma cells, IGF‐1R is rapidly transferred from non‐lipid raft domains to lipid rafts in response to ligand stimulation.^49^, ^50^ In addition to the effect of lipid rafts on the IGF system, the IGF system has an effect on lipid rafts. Studies have shown that in human multiple myeloma cells, stimulation of IGF‐1 leads to the aggregation of lipid rafts on the plasma membrane and the formation of large domains.^51^

## 4 LIPID RAFTS IN THE PI3K/AKT SIGNALLING PATHWAY

Depending on its structure and function, PI3K (phosphatidylinositol 3‐kinase) can be categorized into 3 types, of which class I PI3K is the one most closely related to human cancers. These PI3K types all include a regulatory subunit and a catalytic subunit. Three regulatory subunits, p85α, p85β and p55γ (collectively known as P85), are encoded by the PIK3R1, PIK3R2 and PIK3R3 genes, respectively, and 3 catalytic isomers, p110α, p110β and p110δ, are expressed by the PIK3CA, PIK3CB and PIK3CD genes. In the occurrence and development of human cancer, somatic mutations in PIK3CA and PIK3R1 are common, and the PI3K pathway is activated.^51^ Akt (protein kinase B) has 3 conserved domains, the pleckstrin homology domain (PH), catalytic domain and regulatory domain, in which PH can bind to phosphoinositide with high affinity. There are also 3 subtypes of human AKT, namely, AKT1, AKT2 and AKT3, which are expressed by different genes. Studies have shown that Akt subtypes play unique functions in specific cell lineages and have an important impact on cell physiology. As these 3 subtypes are widely present in almost all cells and tissues, the functional differences of Akt isomers are not exclusively due to differences in their expression levels.^52^, ^53^

As mentioned above, the IGF system can activate many signalling pathways related to physiological activities, such as cell survival, proliferation and division, and the class I PI3K/Akt pathway is a major cell survival pathway.^54^ In response to growth factors such as IGF‐1, the regulatory subunit (P85) of PI3K is recruited to the cytoplasmic domain of activated receptor tyrosine kinases or to phosphotyrosine‐containing sequences in molecules such as IRS‐1. Then, the catalytic subunit (P110) of PI3K binds to and phosphorylates its substrate phosphatidylinositol‐4,5‐diphosphate (P145P2) to produce phosphatidylinositol‐3,4,5‐triphosphate (PIP3), which recruits effector molecules such as Akt to the plasma membrane by specifically binding the PH. This binding induces a conformational change in Akt and phosphorylation of key residues.^55^ After it is fully phosphorylated, Akt maintains its catalytic conformation, is separated from PIP3 and transported to the cytoplasm, nucleus and mitochondria, where it regulates various target proteins and key nodes of downstream pathways by phosphorylating specific substrates, including mTORC1, Bad, GSK3, etc.^55^ Thus, Akt can regulate the gene expression, protein synthesis, cell cycle, cytoskeleton and cell metabolism of cells, making the PI3K/Akt pathway an important participant in the occurrence and development of cancer.^56^ In addition, phosphatase and PTEN on chromosome 10 are negative regulators of the PI3K/ATK pathway, and PTEN can dephosphorylate PIP3, generating PI45P2, which effectively terminates signal transduction.^57^

In fact, among the abnormal pathways in human cancers, the PI3K/Akt pathway is among the most common, and Akt overexpression has been reported to be related to many kinds of cancers, including ovarian cancer, lung cancer and pancreatic cancer.^58^ The overexpression of Akt promotes the proliferation and survival of cancer cells. In addition, the loss of the abundance and function of PTEN leads to excessive accumulation of PIP3, which activates the PI3K/Akt pathway. Therefore, PTEN deficiency is one of the most common types of aberrations in cancer, and its effect is particularly significant in glioblastoma and prostate cancer.^59^ Ediriweera et al.^60^ showed that 10‐gingerol, a natural phenolic lipid, can bind to cell membranes and regulate membrane properties and thus affect the PI3K/Akt signalling pathway in radiation‐resistant triple‐negative breast cancer (MDA‐MB‐231‐IR) cells by regulating lipid raft formation, thereby inhibiting the proliferation, migration and invasion of cancer cells and inducing apoptosis. This process is mainly caused by the entry of amphiphilic 10‐gingerol into the plasma membrane of cancer cells and the transfer of caveolin from the lipid raft to the membrane outside the lipid raft, destroying the lipid raft. Interestingly, in this process, 10‐gingerol did not affect the total expression of caveolin.^60^ Reis‐Sobreiro et al.^61^ showed the important role of lipid rafts and the PI3K/Akt pathway in cancer cell survival. Akt is constitutively activated in MCL cells,^62^ and Akt activation is thought depend on lipid raft function.^63^ After incubating MCL cells with edelfosine, PI3K, p‐PDK and mTOR were displaced from lipid rafts.^61^ Furthermore, edelfosine treatment blocked the PI3K/Akt signalling pathway in MCL cells and induced their apoptosis by entering lipid rafts and replacing Akt, and nullifying the effect of its regulatory factors. After preincubation with pervanadate, MCL cells overcame the apoptosis induced by edelfosine, because pervanadate is an effective Akt agonist that counteracts the cell death induced by Akt level decrease.^61^ These results indicate that the distribution of Akt in lipid rafts is closely related to its metabolic function, which is a key factor in maintaining the normal function of the PI3K/Akt signalling pathway. The relationship between lipid rafts and the PI3K/Akt pathway is critical for cell survival in many tumour cells.

## 5 LIPID RAFTS AND DEATH SIGNALLING PATHWAYS IN CANCER CELLS

Cell death caused by ageing, infection or other injury factors is important for the maintenance of a homeostatic environment in the body. It is particularly important in the process by which the body eliminates mutant cells and prevents the occurrence of tumours. When the DNA repair mechanism fails to reverse a cell mutation, most of the mutant cells undergo programmed cell death, thus preventing cancer.^64^ Resistance to this type of programmed death induction is a basic prerequisite for the further survival and proliferation of mutant cells.^65^ Death receptors in cells play an important role in the process of cell death. These death receptors belong to the tumour necrosis factor (TNF) receptor superfamily (TNFRSF), which includes cell surface receptors highly conserved throughout evolution. The death receptor structure is characterized by an α‐helix of 80–88 amino acid residues in the structural domain of its cytoplasmic domain, namely, the death domain (DD).^30^, ^66^ Death receptors activate apoptosis pathways by binding to their homologous ligands.^67^, ^68^ Eight death receptors have been identified in mammalian cells and can be categorized into four types according to their structural homology: p75 (NTR), tumour necrosis factor receptor 1 (TNFR1), CD95 and TNF‐associated apoptosis‐induced ligand receptor (TRAILR) receptors.^69^ Different death receptors, due to their different amino acid sequences, exhibit diverse structures and functions, with some participating in the immune response or tissue development, reflecting the division of labour among different death receptors, and the CD95 branch is associated with a key function in cell death induction.^70^ In addition, because death receptors and downstream apoptotic signalling molecules are often present within or recruited to lipid rafts in significant numbers, Gajate and Mollinedo^4^, ^10^, ^26^, ^30^, ^71^, ^72^, ^73^ proposed the concept of CASMER (cluster of apoptotic signalling molecule‐enriched rafts). The Fas/CD95 signalling pathway and the relationship between CASMER and lipid rafts in tumour cell apoptosis are discussed in the following sections.

## 6 LIPID RAFTS IN THE FAS/CD95 SIGNALLING PATHWAY

In 1989, Shin Yonehara and Peter H. Krammer discovered an antibody capable of killing certain human cell lines. In fact, the two antibodies recognized the same cell surface antigen. Yonehara named the antigen on the cell surface recognized by this antibody FS7‐associated surface antigen (Fas),^74^ and subsequent studies showed that Fas and its physiologic ligand FasL are the main apoptotic regulators in the mammalian membrane.^75^, ^76^ Antibodies that recognize this antigen are grouped into clusters by CD95 differentiation status. Mature human CD95 is a type I transmembrane receptor that is approximately 45–48 kDa long and consists of 319 amino acids, including a single transmembrane domain containing 17 amino acids, a cysteine‐rich N‐terminal extracellular domain, and a C‐terminal cytoplasmic domain containing 145 amino acids (containing a large number of charged amino acids). Its cytoplasmic domain contains a DD that is homologous to that in other death receptors.^9^, ^66^

**FIGURE 3:**
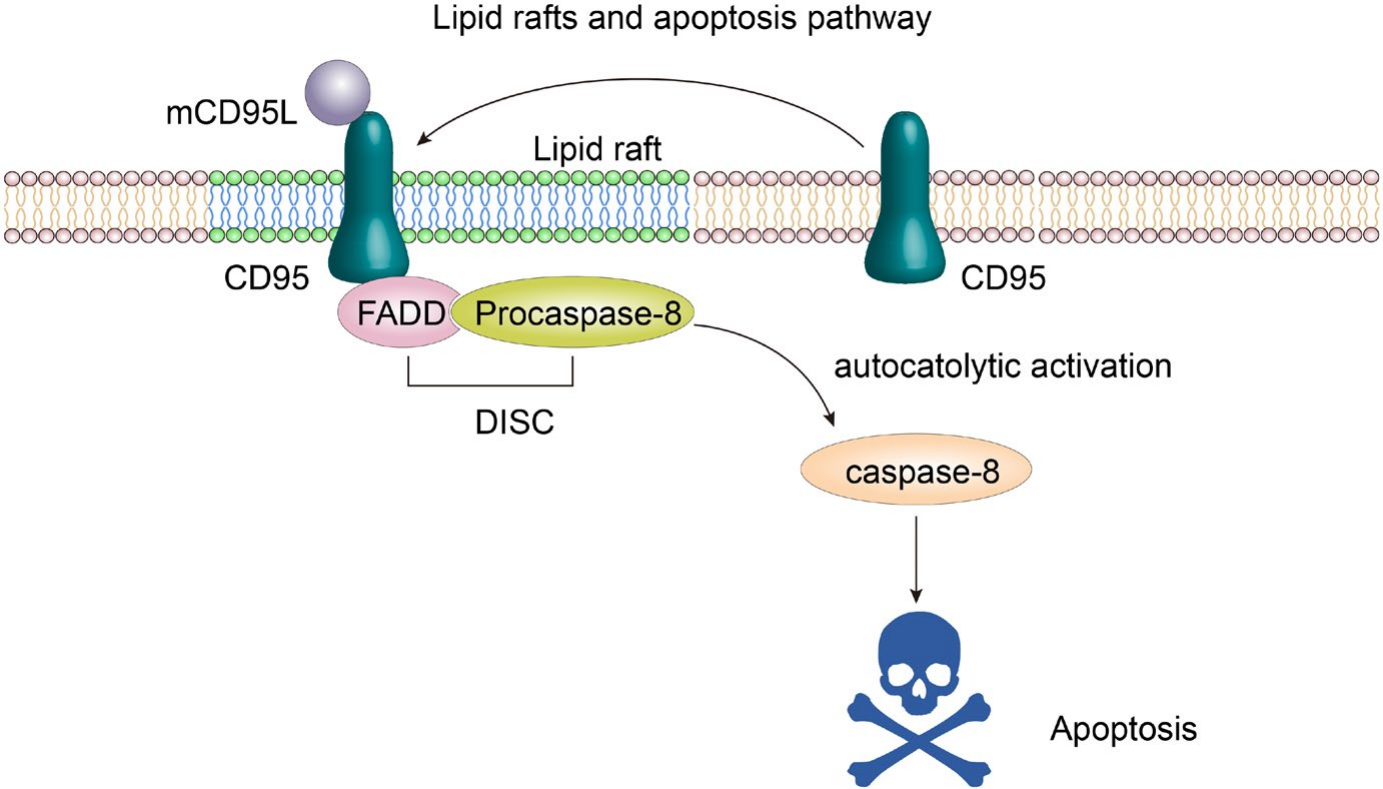
Lipid rafts and the apoptosis pathway. (Simplified from J Lipid Res. 2020; 61: 611–635)

In contrast to the cytoplasmic domains of other transmembrane receptors, the DD has no enzymatic activity but mediates death signalling through protein‐protein interactions. The DD has a tendency to self‐bind and can form larger aggregates in the environment, and the enrichment with charged amino acids may be related to the regulation of the DD.^9^, ^66^ Fas/CD95 binds to its ligand to induce receptor aggregation and recruitment of the connector protein FADD (Fas‐associated protein with death domain) through mutual binding between the receptor and the homotype DD common to FADD, which then binds to the death effector domain of downstream procaspase‐8/‐10, forming the death‐inducing signalling complex (DISC) composed of Fas/CD95 FADD and procaspase‐8/‐10. In fact, due to its low activity, the basic procaspase‐8 enzyme aggregates in the DISC, where individual procaspase‐8 proteins are in sufficiently close proximity to undergo autocleavage, thus initiating a cascade that leads to apoptosis.^26^, ^29^, ^77^, ^78^ Specifically, the direct activation of caspase‐3 through protein cleavage, or the cleavage of Bcl‐2 family protein Bid, triggering mitochondria‐mediated endogenous apoptosis signalling (Figure 3), can occur in parallel to procaspase cleavage.^79^

As early as 2000, CD95 polarization and capping was found in human T cells sensitive to CD95‐mediated apoptosis and human T‐cell leukaemia Jurkat cells treated with edelfosine.^79^ Subsequently, in 2001, Gajate and Mollinedo^3^ found that the recruitment and aggregation of Fas/CD95 death receptors in the plasma membrane lipid rafts of these cancer cells induced the apoptosis of these human acute T‐cell leukaemia Jurkat cells and acute myeloid leukaemia HL‐60 cells upon treatment with edelfosine. After treatment with methyl‐β‐cyclodextrin, the depletion of cholesterol in these cells disturbed the normal structure and function of the lipid rafts, leading to the inhibition of CD95 aggregation induced by edelfosine and the apoptosis signalling pathway that it mediated.^3^ As previously mentioned, lipid rafts are important signalling platforms for the survival and proliferation of tumour cells, and these findings suggest that lipid rafts also play an important role in the regulation of the death signalling pathway as represented by Fas/CD95. Subsequently, Hueber et al.^80^ showed that the natural ligand FasL/CD95L can induce homologous receptor translocation in lipid rafts. Although the above results were similar; however, the mechanism of death receptor aggregation induced by edelfosine and natural ligands was different. Gajate et al.^4^ using microinjection in cells expressing inactive or ectopic Fas/CD95 and unable to take up edelfosine, showed that edelfosine triggered Fas/CD95 activity in cells independently of homologous ligand action. However, the accumulation of edelfosine in the lipid rafts of hematopoietic cancer cells resulted not only in the accumulation of the death receptor CD95 in lipid rafts but also in the promotion of downstream signalling molecule transfer into lipid rafts.^4^, ^5^, ^81^ Other studies have shown that in addition to edelfosine, other antitumor drugs can promote CD95, FADD and procaspase‐8/‐10 entry into lipid rafts to induce the formation of DISCs^5^, ^82^, ^83^ and promote the generation and transmission of downstream apoptotic signals. Similar studies of other death receptors, such as TRAILR, have shown that some antitumor agents can promote intralipid migration of TRAILR and form DISCs with these molecules.^5^, ^82^, ^83^ It is not difficult to see that the recruitment of death receptor signalling molecules such as CD95 to lipid rafts is a novel and promising mechanism in cancer therapy, as originally proposed and advanced by Mollinedo and Gajate's pioneering work.^6^, ^9^, ^26^

Many experimental results have shown that the accumulation and activation of CD95 in lipid rafts can promote the apoptosis of tumour cells. Some studies showed that, in some types of cells, after the depletion of cellular cholesterol by drug treatment, CD95 can spontaneously aggregate in the non‐lipid raft region of the plasma membrane independent of ligand action and combine with FADD and other molecules to promote apoptosis signal transduction.^84^, ^85^, ^86^ These results suggest that CD95‐mediated cell death signalling in tumour cells is indeed regulated by lipid rafts, but the specific mechanism remains to be further clarified. According to the results obtained thus far, these seemingly contradictory conclusions may be the result of differences in cell types.

## 7 LIPID RAFTS AND CASMERS

As mentioned above, when Fas/CD95 functions as a death receptor, it aggregates with FADD, procaspase‐8/‐10 and other molecules in the lipid raft. When the local concentration reaches a certain level, intermolecular interactions trigger death signal transduction. Thus, clusters of apoptotic signalling molecule‐enriched rafts play an important role in death signal transduction, and the term CASMER was created and coined by Mollinedo and Gajate,^84^, ^85^, ^86^ by taking the first letters of each major word that describes this process.^71^, ^73^ CASMER refers to the state where the death receptor and its downstream signalling molecules are recruited to a polymerized lipid raft or raft platform, which is a supramolecular entity dependent on the existence of lipid rafts. Protein molecules interact with each other, and multiple cell death signalling pathways converge here. The CASMER acts as the convergence point and a transmitter of apoptosis signals downstream during cell apoptosis signal transduction.^73^ As a combination of death receptors and multiple death signalling molecules, CASMER protein composition is naturally complex. Recruited death receptors constitute the basic proteins of the CASMER, while the complexity of a CASMER is reflected by the variety and quantity of other downstream signalling molecules, such as FADD and procaspase‐8, components of DISCs and additional proapoptotic molecules, such as Bid and JNK.^10^, ^26^, ^29^, ^30^, ^72^, ^73^, ^87^, ^88^ The unique microenvironment of a CASMER can also modulate the regulatory properties of some of the non‐apoptotic signalling molecules it recruits.^30^

As CASMER carriers, lipids are crucial to the composition and function of a CASMER, which requires both sufficient fluidity to ensure the interaction of protein molecules and a certain rigidity to separate these mixtures. Cholesterol plays a key role in this process, and a lack of cholesterol prevents CASMER formation.^26^ As mentioned above, the cholesterol content of cancer cells is higher than that of normal cells, which may indicate that cancer cells have more potential to form CASMERs, thus providing a new idea for cancer treatment, such as inducing cancer cells to produce CASMERs to activate the death signalling pathway, as pioneerly introduced by Mollinedo's group.^9^, ^10^, ^26^, ^30^

The factors known to be involved in the formation of a CASMER and its protein composition are cell phenotypes and priming stimuli.^9^, ^10^, ^26^, ^30^ For example, most CASMER‐related experimental data have been obtained from hematopoietic tumours, which may indicate that hematopoietic tumour cells are more likely to form CASMERs than solid tumour cells. A series of complete and comprehensive reviews on lipid rafts, CASMERs and cancer have been recently published by F. Mollinedo's group.^26^, ^29^

## 8 LIPID RAFTS AND METASTATIC SIGNALLING IN CANCER CELLS

Metastatic cancer is the deadliest form of cancer because metastases are difficult to excise surgically, and conventional radiotherapy and chemotherapy are often ineffective. Cancer metastases account for 90% of all cancer‐related deaths.^89^ However, few cells survive the process by which a primary tumour is metastasized to a distant organ.^90^ Successful metastasis of tumour cells requires some basic steps including: angiogenesis, epithelial‐to‐mesenchymal transition (EMT), directed migration and transendothelial migration (TEM), survival in circulation, endothelial rolling, extravasation and phenotype switching back to a proliferative state.^90^ Regeneration of the metabolic system of metastatic tumour tissue plays an important role. However, compared with those of aerobic glycolysis and glutamine metabolism, the roles of lipids in cancer metastasis have not been clarified. Lipid rafts, as special selective signal transduction‐related membrane domains, are thought to play special roles in the signal transduction of cancer metastasis.^91^ In the following section, we focus on the roles of the interaction of the VEGF/VEGFR2 axis and CD44 with lipid rafts in cancer metastasis.

## 9 LIPID RAFTS IN THE VEGF/VEGFR2 SIGNALLING PATHWAY

Angiogenesis is the orderly generation of original blood vessels by the regulation of signal molecules, which exist widely under physiological and pathological conditions.^92^ Vascular endothelial growth factor A165 subtype (VEGF) is a key signalling protein in this process and has affinity for the receptor tyrosine kinase on the surface of endothelial cells.^93^ VEGF receptors can be classified into VEGFR1 and VEGFR2. VEGFR1 is thought to inhibit angiogenesis because its kinase activity is impaired and it is unable to mediate downstream angiogenesis. However, VEGFR2 has a complete tyrosine kinase domain, which can activate various downstream signal cascades, leading to permeability change and the migration and proliferation of vascular endothelial cells, thereby mediating the angiogenic effect.^94^

The spatial distribution and function of VEGFR2 are closely related to lipid rafts. In endothelial cells, VEGFR2 has been shown to colocalize with lipid rafts, and they are essential in VEGF‐mediated angiogenesis signalling. Experiments have shown that disruption of lipid raft stability in bovine aortic endothelial cells (BAECs), human umbilical vein endothelial cells (ECV304) and human acute myeloid leukaemia cells (B1647) increased basal phosphorylation and VEGF‐mediated VEGFR2 phosphorylation.^95^, ^96^ Zabroski and Nugent's^97^ study showed that lipid raft destruction selectively reduced the level of inactivated VEGFR2. Three lipid raft inhibitors, methyl‐β‐cyclodextrin, sphingomyelinase and simvastatin, were used to disrupt the lipid raft in the cell membrane through different mechanisms, thereby increasing the lysosomal degradation of VEGFR2, selectively downregulating non‐activated VEGFR2 and leading to reduced activation of downstream ERK pathways.^97^ A study showed that the effect of hepatic X receptor activation on endothelial cholesterol homeostasis impaired VEGFR2 localization to lipid rafts, resulting in loss of VEGFR2 phosphorylation and VEGF‐A‐mediated downstream signalling.^98^ In another study, caveolin‐1, a lipid raft marker secreted by metastatic prostate cancer cells, was found to enhance angiogenic signalling through colocalization and autophosphorylation of endothelial VEGFR2.^99^

In addition to regulating the role of VEGFR2 in cell signal transduction, lipid rafts are also closely related to VEGF secretion from cancer cells. Hsp90 localizes to lipid rafts where it stabilizes CD24, which has been shown to be the basis of STAT3 activation and VEGF angiogenic signalling pathways in colorectal cancer cells.^100^ In addition, lipid rafts also play important roles in cellular exosome uptake, enhancing noncoding miRNA communication between cancer cells and endothelial cells and stroma, which is conducive to angiogenesis.^101^ A study related to ovarian cancer found that endothelial cells internalized exosomal miR‐205 in a lipid raft‐dependent manner, and inhibition of lipid raft‐mediated endocytosis inhibited miR‐205 uptake, thereby inhibiting angiogenesis.^102^

Therefore, the regulation of lipid rafts in tumour cells by means of cellular steroid regulation may be an ideal way to inhibit tumour angiogenesis and prevent of cancer metastasis.

## 10 LIPID RAFTS AND THE CELL ADHESION RECEPTOR CD44

CD44 is a major cell adhesion receptor involved in cell migration and invasion.^103^ CD44 is an 80–95 kDa type I transmembrane glycoprotein, hyaluronic acid is its ligand. CD44 has been shown to play an important role in the signalling of pancreatic and gastric cancer stem cells and is also an important marker for these cancers,^104^, ^105^ because it is highly expressed in many cancers,^106^ and it is involved in the regulation of cancer metastasis. The interaction of CD44 with certain extracellular matrix ligands promotes the process of cell migration and invasion in cancer metastasis. Both the intracellular and extracellular regions of CD44 undergo continuous proteolytic cleavage, causing the release of soluble CD44 from the extracellular region and the dynamic regulation of the CD44 interaction with the ECM during cell migration in the HA matrix during metastasis.^106^

The relationship between CD44 and lipid rafts is complex. Although clear evidence shows that CD44 is located in lipid rafts, the function of CD44 depends on other mechanisms,^107^ primarily because the processing enzymes of CD44, disintegrin and ADAM10, mainly exist in the extracellular lipid raft region, and CD44 must migrate outward from the lipid raft to contact the enzyme processing required for its activity.^108^ Therefore, limiting the extracellular metastasis of CD44 is a possible pathway of cancer metastasis inhibition. CD44 distribution outside the lipid raft of human glioblastoma cells has been shown to induce metalloproteinase‐mediated CD44‐associated cell migration.^109^ Another study with glioma and pancreatic cancer cells showed that the use of methyl‐β‐cyclodextrin to deplete cholesterol in cell membranes and destroy lipid rafts resulted in increased extracellular lipid distribution of CD44 and promoted ADAM‐10‐mediated CD44‐associated tumour metastasis.^106^ In addition, it has been shown that palmitoylation of two cysteine residues in CD44, at positions 286 and 295, in breast cancer cells can result in high affinity of CD44 for cholesterol‐rich lipid rafts, thus limiting the distribution of CD44 to lipid rafts, restricting the binding of CD44 with promigratory binding partners, such as ezrin, and thus reducing the metastatic spread of breast cancer.^110^

All these results indicate that CD44 is an important regulator of cancer cell metastasis and a potential drug target.

## 11 CONCLUSIONS AND FUTURE PERSPECTIVES

Lipid rafts, as cellular structures in tissues that can effectively regulate cell signal transduction, have attracted increasing attention in the academic field since their discovery and have also improved the understanding of the cell membrane in signal transduction. With an increasing number of studies on the mechanism and application of lipid rafts, recent evidence has shown that lipid rafts can promote or inhibit the survival, death and metastasis of tumour cells by selectively recruiting signalling molecules and receptors, which points to the direction of research on the mechanism of cancer occurrence, drug resistance and metastasis and the possible development therapeutic drugs. As mentioned above, relevant basic and clinical studies have shown that the treatment of lipid rafts in the cell membrane and the regulation of lipid metabolism in patients can induce apoptosis and inhibit metastasis. Lipid rafts can protect the receptors residing in them; therefore, the inclusion of survival receptors in lipid rafts may lead to the occurrence of cancer and the generation of drug resistance, and the induction of death receptors in lipid rafts may be a possible way to induce the—apoptosis of cancer cells. In addition to this review on the relationship between CD44 and cancer metastasis, many similar receptor and lipid raft relationships are worthy of investigation. A study showed that CD133, an oncogenic cancer stem cell (CSC) marker, is located in the lipid raft of pancreatic cancer cells and is associated with drug resistance in cancer cells.^111^ In terms of survival and death signalling, the novel proposal of CASMER‐based therapies by Mollinedo's group^10^, ^26^, ^29^, ^30^ appear to have great promise in inducing apoptosis in the treatment of cancer.^112^

However, there are still many questions that remain unanswered. First, the mechanism of lipid raft formation, activation and partitioning remains to be elucidated, and the relationship between lipid raft subtypes and cancer cell types remains unclear. In addition, while a close correlation between lipid rafts and signal transduction has been found, the mechanism(s) by which lipid raft‐mediated signal transduction can be turned on or off remains unknown? Do different lipid rafts interact?

Although many problems remain to be solved, the regulation of lipid rafts remains an important way to regulate the survival, death and metastasis signalling pathways in cancer cells, and these pathways have the potential to be novel targets for cancer therapy.

## CONFLICT OF INTEREST

The authors declare that the research was conducted in the absence of any commercial or financial relationships that could be construed as potential competing interests. The authors have no competing interests to declare.

## AUTHOR CONTRIBUTIONS

BRL and YQ collected the related literature and drafted the manuscript. XJY, XWX and WYX participated in the design of the review and drafted the manuscript. All authors read and approved the final manuscript.

## Data Availability

Data in this study will be shared after approval of the corresponding author on reasonable request.
